# East and West

**Published:** 1887-12-24

**Authors:** Leslie Keith

**Affiliations:** Author of "The Chilcotes," "Uncle Bob's Niece," etc.


### East and West.

By Leslie Keith.

Author of "The Chilcotes," "Uncle Bob's Niece," etc.

(*Continued from p.* 208.)

### Chapter XIII.—Alicia Makes Surrender.

All things come to an end sooner or later, and with the autumn there arrived the last days of Alicia's liberty.

The end—or shall we say the beginning?—had come to David too, in the cottage down by the river, and with his surrender to death there had been a loosening of other ties. The schoolmaster waited for the funeral; they laid the poet to sleep in a quiet place where the voice of London which he had loved was heard remotely, softened and hushed to a murmur, so that you could not distinguish the pain from the pleasure of it; and after the funeral the schoolmaster proposd to carry Mary back with him to her own hills.

"You'll come home wi' me, lass," he said. "While there's bite or sup you'll share it."

But Alicia had forestalled the man of books, and Mary's destiny was already fixed beyond change. The tears were in her eyes when she thanked her old friend, but she said that she could not go with him; she could not leave London—London where her deepest experiences had come to her. "I could not be happy among our bonnie green hills. I have seen the life here; I could not go back, and be at peace."

The Scotchman, being a practical person, then suggested that even if she had the extremely bad taste to prefer the turmoil of the streets to "the sleep that is among the lonely hills," it was still necessary to live.

Then Alicia came to the rescue. "Mary is going to be my ambassador," she said, "the bearer of good tidings. She is good and gentle; the poor people trust her already, and will by-and-bye come to love her, as they would never love me. She is going to do my work—the work I cannot do"—she hung her head—"the work I am not worthy to do." So this proud young lady spoke.

He looked at her shrewdly, and with a gleam of friendliness in the eyes under the shaggy brows. Though she had the misfortune to be English, she was clearly not a bad-hearted lass. He alone had guessed the secret of David's brief success. To the end of her life Mary believed that it was David's genius that had prevailed and won him recognition, and neither Alicia nor Felix Mervyn disturbed that simple faith. But the schoolmaster had gauged his old pupil's limits too accurately to be deceived; he did not betray his discovery, however, being possibly deterred therefrom by a certain defiance in Alicia's eyes. He was also too wise to attempt the arrest or overturn of the plans of two such determined young women. So he took his homeward way alone, with a copy of David's poems in his pocket to beguile the tedium of the journey.

There came after this the other parting on the day when Alicia took farewell of the top room in Baron Street and of Mary, and went back to Park Lane and greatness.

Her family received her variously. Her father found her grown more beautiful; Kitty looked at her with something between respect and awe, and—pity, shall we say? There were moments when Kitty felt herself immensely superior to her elder; it was her private consolation for many snubs. Alicia, however, no longer snubbed. She went about as one who dreams, waking up when Felix Mervyn came to call, as he sometimes did. On those visits they held the talk mostly to themselves, the others suffering the embarrassment of listening, as it were, to an unknown tongue; but when the doctor left the drawing-room it was noticed that he was frequently enticed into the study, where, it may be presumed, he and Mr. Frankland did something more than smoke in silence.

After a while he so far consented to be disinterred as to accept an invitation to dinner, and having come once, there seemed to be some excuse for coming again. It was after one of those family meals that Alicia had an inspiration—what our American allies would call a bright idea. She matured this plan—for it speedily became a plan—in secret, and thrust it upon her family only when she was so enamoured of it as to be quite safe from conversion by argument.

Her fancy was to summon the scattered members of her set whom November had brought back to town. The autumn rigours had driven society from country houses, whose dulness the wet season had rendered insupportable. London was dull also. Therefore Alicia's great party fell upon a happy moment. When people are bored and have nothing to do an invitation awakens a languid gratitude.

It was not a brilliant resource, as she was the first to acknowledge, but it was the best she could contrive. She took the greatest possible pains with her arrangements; she asked everybody she could think of, and everybody accepted with alacrity; she insisted that her father should produce the rarest vintage from his cellar; she ransacked all the quarters of the globe to provide a supper; her own dress was a miracle of skill and taste.

Kitty looked on awhile with round eyes, and then she said to her lover—

"Do you know what Alicia is going to do, Jack? She is going to talk to the people." Then she went on with decision: "We mustn't be behind; we must do our part."

"But, I say, we can't talk, you know," said Jack, looking rather blank.

"Well, I don't know." Kitty did not seem willing to yield the point. "But, anyhow, when we go down to live at Highmead we must have gutter-parties."

"All right," said Jack, with a lover's complacency; "we'll set a wing of the old barrack apart for the use of decayed ladies and gentlemen, if you like."

"And you will give them moral instruction in the summer evenings, Jack. You will make an excellent teacher—with practice."

"All right," said the obedient lover once more, but this time with a hint of reluctance.

This was indeed the manner in which Alicia proposed to amuse her guests. She meant to talk to them of that strange land—strange to all of them as an undiscovered America—where her summer days had been spent.

"I will tell them about it," she said to her father and Mervyn; "perhaps they will listen to one of their own set. I will tell them about the sick people who have to be turned away from the hospitals, not for lack of room, but because we, who are able, refuse the little sum that will keep them there. Perhaps that will touch them. There is a good deal of gout (induced by too much lobster and champagne) among my audience, and every imaginable form of 'nerves'; perhaps a fellow-feeling will make them kind. I will tell them about the sewing-girls—the *men* will pity them," she said, with a touch of cynicism; "about that child who went to school with the cover of an old umbrella for her sole garment (Mrs. Livingston-Lightly pays five pounds for each of her children's summer cottons); about their homes—our own will emphasise the moral. There is that poor old cobbler who has seen generation after generation of the miserable come and go, and who, in the thirty-three years he has lived there has had his single room papered once and painted not at all. When he first went there with his wife the whole house was let as a place of storage for five shillings a-week; not a room in it now fetches less than four shillings—and such rooms! The poorest and humblest of our servants would not sleep a night in one of them. "Oh," she cried, "there is so much to say, if I can only make them listen."

"They will listen," said Mervyn with deeply-moved conviction, "they couldn't help it if they would."

He was eager in aiding her with details and illustrations.

"I mustn't make a statement that I can't support with proof," she said. "I have the fear of my lawyer-guests upon me. Suppose they cross-examine me?"

To the last, however, Mervyn doubted whether he should be present. He had fallen out of the ranks of society, and laughingly declared he should scarce know how to behave. "They will take me for a specimen of the Eastern aborigines," he said lightly; he was ready with a hundred excuses, fearing, perhaps, the risk to his peace if he yielded.

But when Alicia looked at him and said simply, "You will come?" he had no other answer but "Yes."

The evening of the great event found Alicia alone for a moment with her father in the library. Mr. Frankland had grown bold in these last days, and no longer refrained from demonstration. His hand was on her shoulder, he looked into her flushed and kindled face, and his question seemed almost superfluous:

"Are you sure you have the courage to carry this out?"

"Quite sure," she said bravely. "To have seen what I saw, would give anybody courage."

"And—afterwards? Have you faced the consequences, my child? Do you know to what you are committing yourself?"

Alicia's head drooped, and this time her voice was tremulous. "I think so," she said.

"What shall I do without you?" he asked rather sadly.

"I shall not be lost to you, papa, and"—she looked up with a brave attempt at lightness—"you will smoke the more!"

The guests now began to arrive: lords and ladies and untitled, but still highly genteel folks; ladies of all conditions and ages—the bored, the sprightly, the frivolous, the languid. Men in plenty, too; Alicia's phalanx and Kitty's chosen battalion, and those also who were supposed—being appropriated by neither sister—to come for Mr. Frankland's sake.

Well-bred was the surprise of this vast audience to find the handsome reception-rooms seated as for a lecture. Who was the lecturer, and what was his subject? It was a new departure for the Franklands, who were usually rather original. Then the depressed among the company began to console themselves. It was certain to be some amusing novelty, or Alicia Frankland would not have countenanced it—she whose greatest enemy was boredom.

Alicia had received the company with a charming grace, and when, taking her father's arm, she moved to the head of the room and took her place behind a little table furnished with nothing but a glass of water, there went through the ranks a murmur of astonishment.

Perhaps the first thought present in most minds was that she had, as her father had said, grown more beautiful. The sleeping depths were stirred; the slumbering soul had awakened, and divine love and tenderness shone in the eyes that had looked with pride and haughtiness on all the world. A young man, unrecognised by the gay company, and occupying a remote corner, felt his heart go out to her with a great ache and tenderness. "She is an angel," he said to himself; "how shall we live without her?" Her father's fear lest she should break down was soon dissipated. She did not break down. She clasped her hands, and looked at the sea of faces before her. "We have all been wandering during the summer time," she began. "Not one of us, I suppose, has remained here. I have been away too—on a strange journey among a strange people. If you will allow me—if you will have patience with me, I should like to tell you something of what I saw and heard there."

There was a little murmur of encouragement and interest, and, after a faltering sentence or two, she forgot everything but the story of sad, unfriended toil and misery she had to tell, and she told it with an absence of self-consciousness and an earnestness that gained her every ear. A supreme belief in one's cause, that is the secret of influence. Alicia's lecture shall not be given here. The facts she cited are no longer new—or ought not to be, for many other explorers have come back to tell her story, and perhaps if an Alicia of to-day were to talk of the wrongs of the poor, of their loveless, unamused lives, their shameful deaths, their pains and woes and griefs and sins, she might speak to unheeding ears.

Did our Alicia do any good? Perhaps her burning words touched a generous heart here and there—a young heart, as yet not quite hard. As for the old, the eloquence is not yet discovered that will convert them; they can at best but be moved to a show of generosity.

When Alicia touched on the hospital question she did not spare. "It is we who have emptied those beds," she said. "It is we who have turned the sick and the miserable away from their only refuge; it is we who have decreed that they shall die in their wretchedness. We all of us know what it is to be ill; we should think it very hard if in our pain we were denied the sympathy of our friends; and yet what do we give to those others—our brothers and sisters—in their extremity? Not even a bed to die in! There is a way to remedy this fault, to wipe away this stain; it is a very easy way—if it were difficult we should not take it. It is not our time that is asked, or our services—only our money. Shall we refuse it?"

This naturally made some people a little uncomfortable. Nobody likes to be called a murderer, and consequently there was a good subscription for St. Placida's. Alicia had devised rather a cunning scheme to secure this. She knew very well that ladies, at least, do not come to evening parties with their purses in their pockets, and she was too experienced to trust to promises. Consequently she had provided a dainty little card and pencil for each guest, on which he and she was invited to put down the amount he was willing to give, with the assurance that it should be called for on the following day. A number of young people were told off to distribute the cards, and to secure the name and address of each subscriber. The cunning of the device lay in this, that no one knew what his neighbour was giving. When you are supported by the knowledge that Sir Harry has compounded with his conscience for five shillings, and Lady De B. been generous to the tune of ten, your own modest subscription does not shame you; but suppose Sir Harry has given his pledge for five pounds, and Lady De B. for twenty, how will your seven and sixpence look then? The motive is not high, but our motives are frequently a queer compound.

After the lecture there was much airy comment and exclamation, and many eye-glasses were put up to examine the specimens of native produce Alicia had brought back from her travels; the dolls, match-boxes, what-nots, made by her poor friends—bits of needlework, too, and other things, which were voted "so delightfully ugly, you know." They crowded round Alicia with many questions.

"And did you really go and live down there? So brave of you; but weren't you horribly afraid?"

"Of what should I be afraid?" asked Alicia calmly. "They are men and women like ourselves—only some of them are better than we."

This raised a little murmur of shocked dissent. "And that cabman—did you actually sit with him while he told you the story of his life? It's odd, you know, but somehow when one hails a hansom it's the horse one looks at and not the man."

"Perhaps now that you have heard how he spends his time while we are dancing, you will sometimes think of the man, too," said Alicia gently, addressing the boy who had spoken. She answered all their questions, but it was those who did not ask any of whom she had most hope. Finally there was supper to console the guests, who found it the best part of the entertainment. They went away for the most part rather mystified, and not quite sure whether Alicia had not been practising her satire upon them, and working on their feelings for her own amusement. "It was really too eccentric, her going down there; I wonder her father allowed it," said Society, shaking its head over this unconventional proceeding. Then some one suggested that perhaps she did not go at all, and this view found many to favour it. Perhaps she did not go at all—perhaps the dreadful things she had told them did not exist. Perhaps there was no East-end. The doubt made them feel a little less uncomfortable for what was left of the night, and by the next day, in the minds of all but a few, something else had crowded out Alicia's story.

When the last of her guests had left, Alicia found herself alone with her coadjutor and assistant.

"We can re-open that closed ward," he said, with professional joy. "It is you who have done this. Will you let me thank you before I go?"

Alicia looked very pale and weary. "Must you go?" she said. He took a step forward. "The question is, Must I go alone?" he said.

"Oh," said Alicia, shrinking back, "I don't think I am good enough for the work. I haven't the way. The people would resent my attempts to help."

"The people!" he said, with a most natural vehemence. "It is myself I am thinking of. It is I who want you—I who cannot do without you!"

A little smile crept into Alicia's eyes at that. "If I could really help you—you who are doing the great work—that would make it seem different."

"Yes," cried the young man with a joyous laugh, "it would make it seem different!"

By and by, however, Alicia seemed to feel the need of a lighter tone.

"We have both of us tried the West for a quarter of a century," she said, "it is only fair we should give the same trial to the East. By the end of that time we shall be old enough and wise enough to choose; and if you prefer it we shall return to the world of society. You shall make a specialty of the fashionable complaint of the day, and grow sleek and smug in a West End square, and the Queen—or it may be the King then—will make you a baronet. Being only a doctor, you musn't presume to look for any higher reward."

To this Felix Mervyn had a very obvious reply. "I have got my reward beforehand," he said.

THE END

